# Spinal Hydatidosis in the Lumbar Region: An Unusual Presentation of Echinococcosis

**DOI:** 10.7759/cureus.96062

**Published:** 2025-11-04

**Authors:** Omar Lamridi, Yassine Akrim, Othmane Naouis, Awatif El Hakkouni

**Affiliations:** 1 Parasitology-Mycology Laboratory, Mohammed VI University Hospital, Marrakech, MAR; 2 Biology Department/Parasitology-Mycology Laboratory, Faculty of Medicine and Pharmacy, Cadi Ayyad University, Marrakech, MAR

**Keywords:** albendazole, echinococcosis, echinococcus granulosus, lumbar spine, parasitic infection, spinal hydatidosis

## Abstract

Spinal hydatidosis is a rare and often overlooked manifestation of echinococcosis caused by *Echinococcus granulosus*. We present the case of a 35-year-old female patient from a rural area who presented with progressive lower back pain and heaviness in the lower limbs. Magnetic resonance imaging (MRI) revealed multiloculated cystic lesions at the L3 and L4 vertebrae, suggestive of hydatid disease. The patient underwent surgical excision of the cysts and spinal stabilization, along with albendazole therapy administered pre- and postoperatively. The parasitological examination of the surgical specimen confirmed the diagnosis. The postoperative course was uneventful, and no signs of recurrence were observed at the three-month follow-up. This case underscores the importance of including spinal echinococcosis in the differential diagnosis of vertebral lesions, particularly in endemic regions and among patients with risk factors such as close contact with dogs. Early diagnosis through imaging and a combined surgical-medical treatment approach are crucial for favorable outcomes.

## Introduction

Echinococcosis in humans is a zoonotic disease caused by the parasite *Echinococcus granulosus*, which develops into larval cysts within human organs. This tapeworm resides in the intestines of canines, primarily dogs, which serve as its definitive hosts. The parasite's eggs are excreted in the feces of infected dogs and may be ingested by intermediate hosts such as sheep, cattle, goats, pigs, or, inadvertently, humans. Hydatid disease remains a significant public health concern, especially in regions where sheep farming is widespread, such as Morocco [1].

The liver, lungs, and brain are the organs most commonly impacted, whereas bone involvement is uncommon and reported in only 0.5%-2% of cases. Lumbar vertebral involvement is extremely rare. The most common occurrence is the dorsal level [2].

We report the case of a 35-year-old woman diagnosed with a lumbar vertebral hydatid cyst, who presented with lower back pain and a sensation of heaviness in the lower limbs.

## Case presentation

We present the case of a 35-year-old woman from a rural area with no significant medical history, who was admitted to the emergency department due to progressive lower back pain and a sensation of heaviness in the lower limbs that had developed over the past seven months. Her general condition was preserved at presentation. Upon questioning, it was noted that she had a history of frequent contact with dogs.

On initial examination, the patient was hemodynamically stable, with normal temperature, heart rate, blood pressure, and oxygen saturation. Neurological examination revealed no abnormalities.

Laboratory findings were unremarkable, with normal hemoglobin (15.1 g/dL), white blood cell count (8,610 cells/mm³), and C-reactive protein (2.5 mg/L). Liver function tests, renal profile (creatinine and urea), electrolytes, and coagulation parameters were all within normal limits.

**Figure 1:**
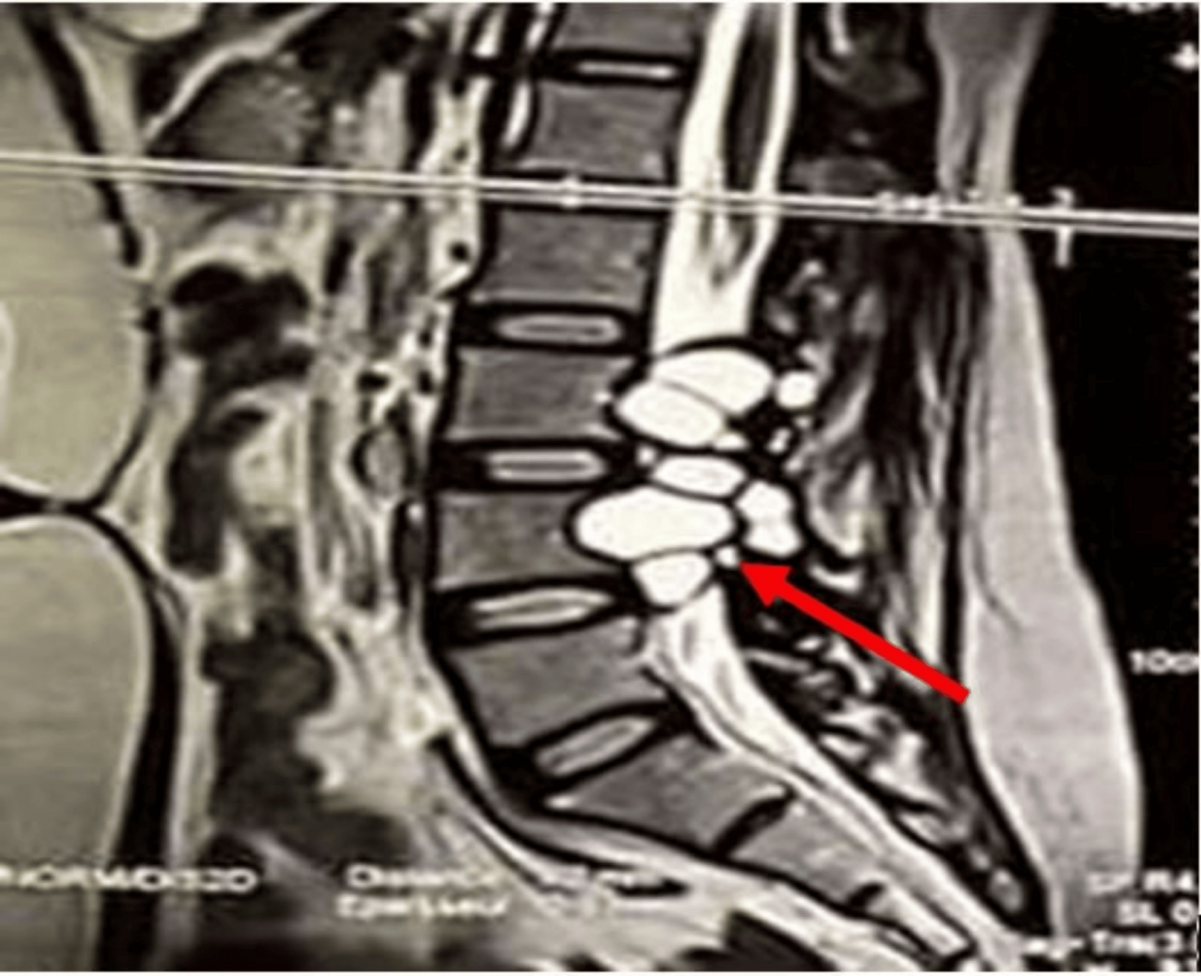
Lumbar MRI sagittal image showing multiple cystic lesions (red arrow). MRI: magnetic resonance imaging

Spinal magnetic resonance imaging (MRI) revealed two well-defined, multiloculated cystic lesions with thin walls located at the levels of L3 and L4. These measured 34 × 32 mm and 36 × 35 mm, respectively, and were associated with vertebral scalloping and canal narrowing at the corresponding levels, findings highly suggestive of lumbar spinal hydatidosis (Figure 1).

Preoperative treatment included albendazole at a dose of 10 mg/kg/day, divided into two doses, administered for two days prior to surgery. The patient underwent surgical removal of multiple cystic vesicles, followed by spinal stabilization using osteosynthesis.

**Figure 2:**
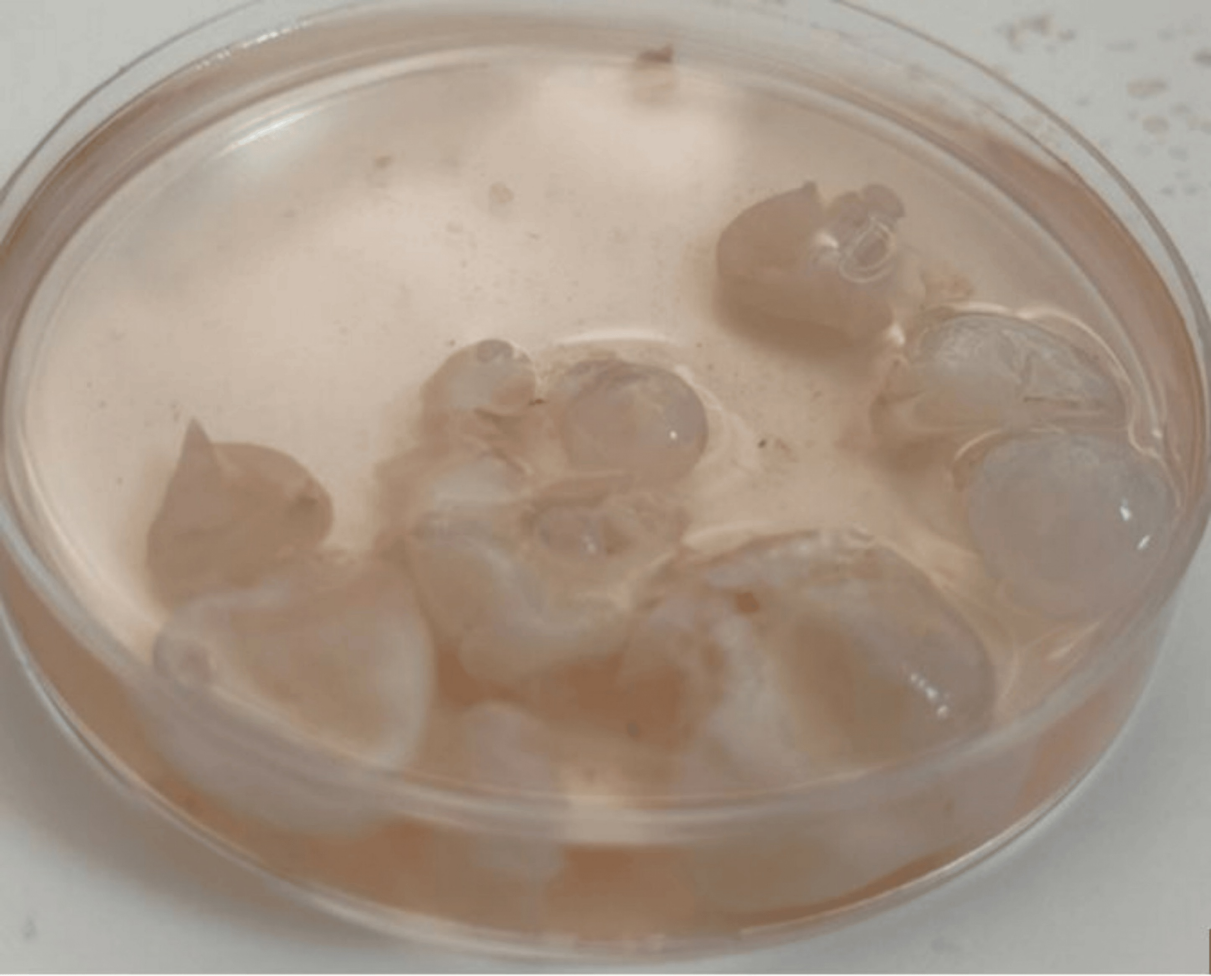
Macroscopic findings of extracted hydatid cysts.

The excised material was sent to the parasitology department for analysis. Macroscopic examination revealed a pearly white, spherical cyst filled with clear fluid (Figure 2).

**Figure 3:**
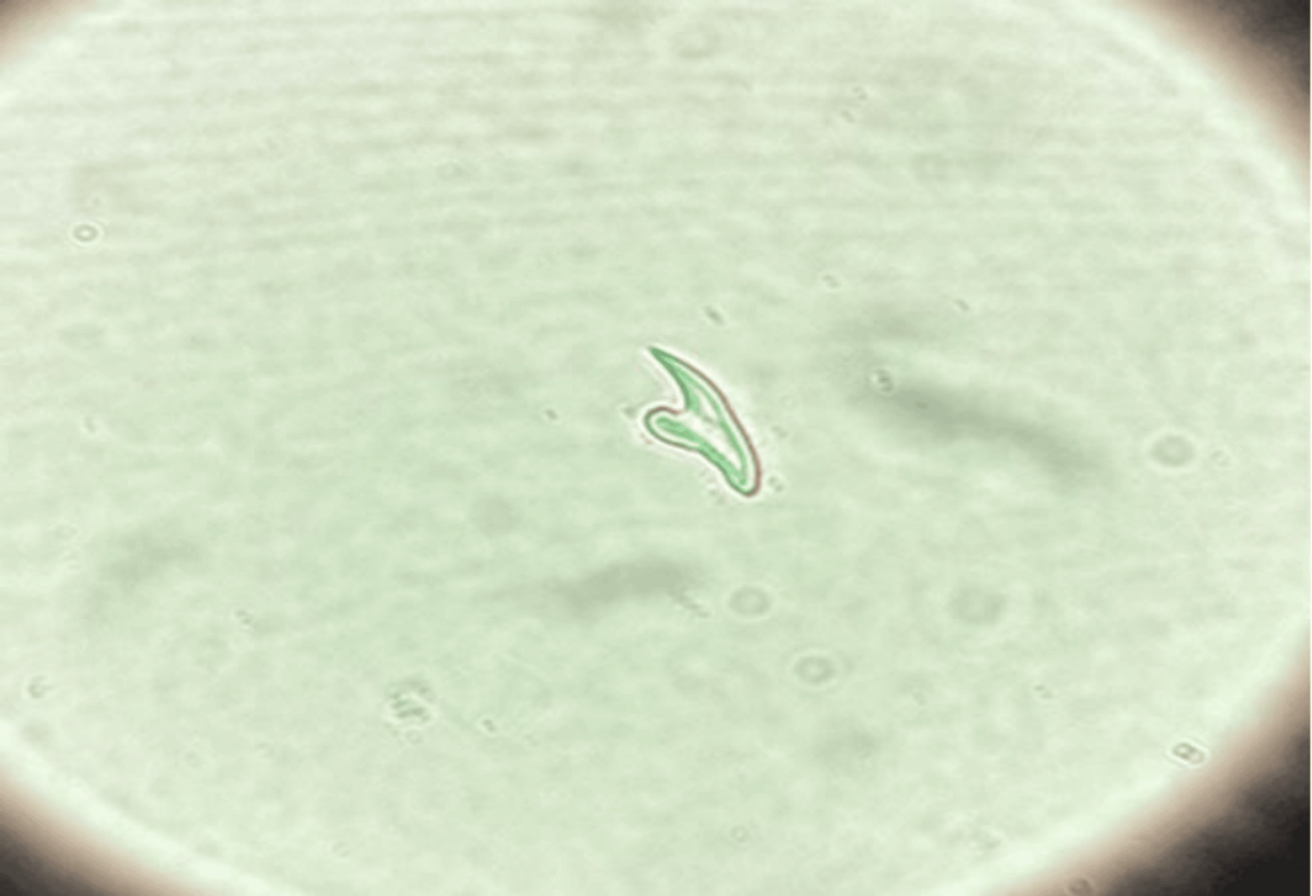
Microscopic examination (40× magnification) revealed a free hooklet within the hydatid cyst fluid.

Microscopic analysis confirmed the diagnosis by identifying multiple protoscoleces and the free hooklet characteristic of *Echinococcus* species (Figure 3).

Postoperative albendazole therapy was resumed at the same dosage and continued on a long-term basis. The postoperative course was uneventful. At the three-month follow-up, the patient reported no back pain or neurological symptoms. There were no signs of recurrence, and she continues to be monitored closely.

## Discussion

Human echinococcosis is a parasitic infection caused by *Echinococcus granulosus*, endemic in regions with extensive sheep farming, such as Morocco [1]. Although the liver and lungs are the most commonly affected organs, osseous involvement remains rare, occurring in 0.5%-2% of cases, with vertebral hydatidosis representing only a fraction of these [2]. The thoracic spine is the most frequent site in spinal hydatid disease, making lumbar involvement, as seen in our case, extremely uncommon [3].

Clinically, spinal hydatid disease presents with nonspecific symptoms such as back pain, stiffness, or neurological signs due to spinal cord or nerve root compression [4]. In our patient, the presentation was limited to mechanical back pain and heaviness in the lower limbs, with no neurological deficit. This aligns with the often slow and silent progression of spinal hydatid cysts, which can delay diagnosis [5]. The patient's rural background and history of regular contact with dogs increased her risk of exposure and infection [1].

MRI is the gold standard imaging modality for spinal echinococcosis, as it provides excellent soft tissue contrast and defines the extent of the lesion [6]. In our case, MRI revealed multiloculated cystic masses at L3 and L4 with vertebral scalloping and canal narrowing, consistent with vertebral hydatid disease [6]. Laboratory investigations, including inflammatory markers, are usually nonspecific and often within normal ranges, as observed in our patient [7].

Treatment involves a combination of surgical and medical approaches. The surgical excision of the cysts aims to decompress neural elements and stabilize the spine when necessary [8]. Albendazole, administered before and after surgery, is recommended to reduce the risk of recurrence and manage potential residual disease [9]. Our patient underwent successful surgical removal, followed by spinal osteosynthesis, and albendazole was continued postoperatively at the standard dose [9].

Prognosis depends on the extent of disease and the completeness of surgical excision. Recurrence is possible, particularly in cases of incomplete removal or spillage of cyst contents [10]. At the three-month follow-up, our patient remained asymptomatic, with no evidence of recurrence, underscoring the importance of early diagnosis, appropriate surgical intervention, and antiparasitic therapy [10].

## Conclusions

Spinal hydatidosis, particularly in the lumbar region, is an extremely rare manifestation of *Echinococcus granulosus* infection and presents a diagnostic and therapeutic challenge due to its insidious progression and nonspecific clinical features. Early imaging, especially MRI, is crucial for timely diagnosis and appropriate surgical planning. A combined approach involving surgical excision and prolonged antiparasitic therapy with albendazole remains the cornerstone of management. Hydatid disease should be considered in the differential diagnosis of spinal lesions, particularly in endemic regions and among patients with risk factors such as close contact with dogs. Long-term follow-up is essential to monitor for recurrence and ensure favorable outcomes.
